# Making the connections: physical and electric interactions in biohybrid photosynthetic systems

**DOI:** 10.1039/d3ee01265d

**Published:** 2023-08-16

**Authors:** Ying Yang, Lu-Ning Liu, Haining Tian, Andrew I. Cooper, Reiner Sebastian Sprick

**Affiliations:** a Materials Innovation Factory and Department of Chemistry, University of Liverpool Liverpool L7 3NY UK aicooper@liverpool.ac.uk; b Institute of Systems, Molecular and Integrative Biology, University of Liverpool Liverpool L69 7ZB UK; c College of Marine Life Sciences, and Frontiers Science Centre for Deep Ocean Multispheres and Earth System, Ocean University of China 266003 Qingdao P. R. China; d Department of Chemistry-Ångström Laboratories, Uppsala University Box 523 751 20 Uppsala Sweden; e Department of Pure and Applied Chemistry, University of Strathclyde Thomas Graham Building, 295 Cathedral Street Glasgow G1 1XL UK Sebastian.sprick@strath.ac.uk

## Abstract

Biohybrid photosynthesis systems, which combine biological and non-biological materials, have attracted recent interest in solar-to-chemical energy conversion. However, the solar efficiencies of such systems remain low, despite advances in both artificial photosynthesis and synthetic biology. Here we discuss the potential of conjugated organic materials as photosensitisers for biological hybrid systems compared to traditional inorganic semiconductors. Organic materials offer the ability to tune both photophysical properties and the specific physicochemical interactions between the photosensitiser and biological cells, thus improving stability and charge transfer. We highlight the state-of-the-art and opportunities for new approaches in designing new biohybrid systems. This perspective also summarises the current understanding of the underlying electron transport process and highlights the research areas that need to be pursued to underpin the development of hybrid photosynthesis systems.

## Abstract 2

Biohybrid systems of synthetic materials and microorganisms can be obtained using a range of assembly strategies based on their interactions. This influences charge transfer between the components and their efficiency for solar fuels generation.

## Graphical abstract

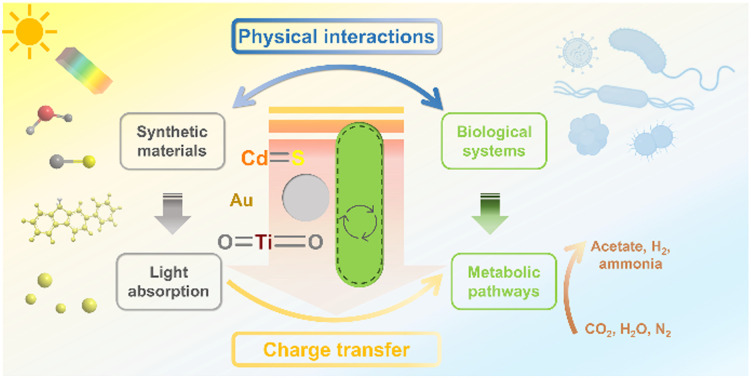

**Broader context**

The basic aim of biohybrid photosynthesis is to combine the efficient light-absorbing properties of synthetic materials with the efficient metabolic pathways in biological systems to convert sunlight into solar fuels or high-value chemicals. This area has attracted significant interest, but our knowledge of how to couple synthetic materials with microorganisms, and the underlying energy transfer processes, is still rudimentary. This perspective discusses the possible assembly principles of biohybrid systems, with a special focus on photocatalyst material-based biohybrid systems, and the associated energy transfer mechanisms. We also highlight new research opportunities that have arisen in recent years.

## 1. Introduction

Increasing global energy demands force us to seek environmentally sustainable alternatives to fossil fuels.^1^ Solar energy is a renewable energy source that could address this need over the next century.^2^ The use of solar energy requires solar capture/conversion and storage; chemical fuels are one sustainable solution to energy storage.^3^ A wide range of potential fuels can potentially be generated including hydrocarbons, nitrogen-based fuels, and hydrogen.^4^ Beyond the direct use as fuels, new solar-derived chemical building blocks can also play an important role in the chemical industry, which is still heavily dependent on non-renewable feedstocks.^5^

Over billions of years of evolution, photosynthetic organisms have developed the photosynthetic machinery to capture sunlight and convert it into organic molecules (*i.e.*, biomass) to store solar energy in the form of chemical bonds.^6–8^ Natural photosynthesis is relatively inefficient compared to the potential thermodynamic limit; a maximum photosynthetic efficiency of about 4.5% has been calculated by Thorndike.^9^

It has been a long-standing challenge to develop practical artificial photosynthetic systems and many different approaches have been used such as photoelectrochemical systems,^10^ photovoltaic cells,^11^ and photocatalyst materials.^12,13^

Artificial photosynthesis mimics these biological systems by using solar energy to drive a thermodynamically uphill reaction to generate fuels.^14^ Photogenerated charge carriers are used for hydrogen production from water or to produce other organic molecules, such as methanol *via* CO_2_ reduction.^2^

Typically, the solar-to-hydrogen efficiency is much lower than those of photosynthetic organisms,^6^ even though solar light absorption process can be more efficient in artificial semiconductors. Biological systems also have numerous advantages of over artificial photosynthesis, including the ability to continually and selectively generate active multi-complex macromolecules and to facilitate electron transfer, as well as sustainable repair and physiological regulation.^28^ Hence, to enhance solar-fuel conversion efficiencies, different strategies have been employed to interface synthetic and biological components. This has prompted researchers to explore artificial photosynthetic systems that are inspired by natural photosynthetic machinery, with the aim of combining the advantages of biological systems with carefully designed semiconductors. This relatively new approach is often termed semi-artificial photosynthesis or biological-chemical hybrid photosynthesis.^28^

In these hybrid systems an irradiated material captures solar energy, generates individual charges and transfers photoexcited electrons to biocatalysts, including enzymes and microbes, to produce hydrogen or other products. Progress has been made in this field since the early 1980s when scientists began to combine inorganic semiconductors with microorganisms to increase hydrogen production,^29^ but semi-artificial photosynthesis has only been studied more extensively since the beginning of this century. A range of systems that capture sunlight can be coupled with biological systems such as photovoltaics,^30^ photoelectrodes,^31^ and photocatalysts,^32^ thus allowing the construction of diverse hybrid photosynthetic systems. Most studies report either hydrogen or acetate as their product (Table 1). The crucial goal of biohybrid photosynthetic systems is to transform essentially inexhaustible atmospheric CO_2_, N_2_, or even wastewater into high-value chemicals with high yield and selectivities,^31,33^ and in the longer term, with high catalyst stability.

**Table 1:** Summary of selected cell/photosensitizer-based biohybrid systems for semi-artificial photosynthesis

Material	Size and morphology	Microorganism	Product and activity	Ref.
CdS	<10 nm; NPs	*M. thermoacetica*	Acetate, 0.48 mM day^−1^, QE: 2.14 ± 0.16%	15
CdS, TiO_2_–MnPc	<10 nm; NPs	*M. thermoacetica*	Acetate, 1.2 mM day^−1^	16
TiO_2_	Anatase powder	Engineered *E. coli*	Hydrogen, 0.72 μmol min^−1^ (mg wet cell)^−1^	17
Water-soluble dyes, inorganic complexes	n.d.	*S. oneidensis*	Activity for several processes: H_2_ production, fumarate and pyruvate reduction, CO_2_ reduction	18
CdS	15–20 nm; NPs	*E. coli*	Hydrogen, >1.8 mmol over 3 hours	19
CdS	<50 nm; NPs	Engineered *E. coli*	Hydrogen, 13.4 μmol after 6 hours, 81.8 μmol after 24 hours (10^8^ cells)	20
AuNCs	Nanocluster	*M. thermoacetica*	Hydrogen, after 24 hours overall QE: 2.86 ± 0.38%	21
PFP/PDI	n.d.	*M. thermoacetica*	Acetic acid, 0.63 mM accumulated over a 3 day experiment, QE: 1.6%	22
CdS	4.1 ± 1.4 nm; NPs	*S. oneidensis MR-1*	Hydrogen, 362.44 ± 119.69 μmol mg^−1^ produced over a total of 72 hours	23
CdS/CsgA_A7_	Nanofibers	Engineered *E. coli*	Formic acid, 0.84 mM within 8 hours, QE: 0.13%	24
PFTP–PSMA D–A CPNs@threonine deaminase	93.2 ± 8.2 nm; NPs	Engineered *E. coli*	2-Oxobutyrate, 6.0 ± 0.15 mM cumulative over 72 hours	25
La/Rh co-doped SrTiO_3_, Mo-doped BiVO_4_	Monolithic photocatalyst sheets	*Sporomusa ovata*	Acetate, solar-to-fuel conversion efficiency of 0.7% at ambient conditions (298 K, 1 atm)	26
PFODTBT polymer dots	Average size 70 nm; polymer dots	*Ralstonia eutropha H16*	Poly-3-hydroxybutyrate, a yield of 21.3 ± 3.78 mg L^−1^	27

Although research programmes in artificial photosynthesis and synthetic biology are moving the field of semi-artificial photosynthesis forward, many of the fundamentals are still under investigation. In particular, the design principles underpinning the construction of functional hybrid systems, and an in-depth understanding of the extracellular/intracellular electron transfer pathways has not been achieved, even though both are crucial steps in these hybrid systems. A central problem here is that the interfaces between the biological and synthetic materials are often not well understood.

## 2. Assembly principles of material–microorganism complexes for hybrid photosynthesis

**Fig. 1:**
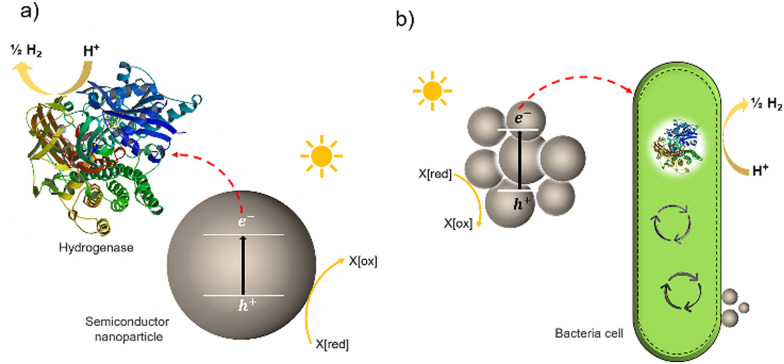
Two typically studied semi-artificial systems. (a) H_2_ production with enzyme hybrids consisting of semiconductor NPs and [NiFeSe]-hydrogenase (PDB ID: 1CC1).^28^ (b) Cell hybrid semi-artificial photosynthesis system. Irradiation of NPs results in the generation of excitons, separation of the excitons, and electron transfer to hydrogenase or cell with H_2_ formation and hole quenching in the nanoparticle. There are multiple potential charge-transfer mechanisms from semiconductors to microorganisms.

Much effort has been devoted to interfacing biological components, such as enzymes and cells, with inorganic and organic materials for photochemical conversion. Two classes of semi-artificial systems are common: enzyme hybrids (Fig. 1(a)) and cell hybrids (Fig. 1(b)); each has limitations and strengths.^28^ Examples of enzyme hybrids include direct immobilization of enzymes onto electrodes, *e.g.*, [FeFe]-hydrogenase adsorbed onto a carbon electrode in a dye-sensitized photoelectrochemical cell,^34^ and nanoparticulate semiconductors ([NiFeSe]-hydrogenase adsorbed to particulates such as dye-sensitized TiO_2_,^35^ [FeFe]-hydrogenase adsorbed polymer nanoparticles).^36^ While their poor scalability and inherent instability render enzyme hybrids impractical for commercial applications,^37^ these studies have advanced the fundamental understanding of these systems and aid in understanding the potential of future applications in cell hybrids.

**Fig. 2:**
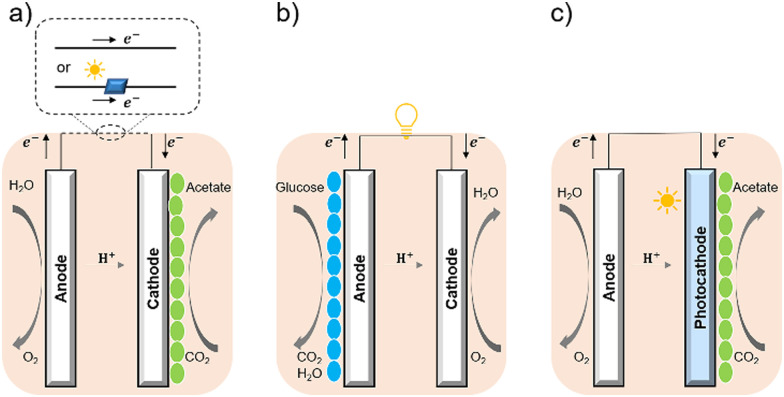
Configuration of (a) MES,^35^ (b) MFC,^36^ and (c) PBEC.^37,38^ (a) MES applies current to the microorganisms (green ellipse) on the cathode to stimulate microbial metabolism. (b) MFC extracts current from the microbial (blue ellipse) metabolism from the anode, analogous to chemical batteries. (c) PBEC derived from photoelectrochemical cells can include a photoanode, a photocathode, or both. Solar energy captured by light absorbers and used by microbial catalysts (green ellipse) to reduce CO_2_.

Bacterial cells have been used as biocatalysts to overcome the limitations of enzymes in the field of semi-artificial photosynthesis. The interactions between microorganisms and electrodes have been reviewed, such as microbial electrosynthesis (MES) reactors^38^ in conjunction with or without solid-state photovoltaic (PV), microbial fuel cells (MFC),^39^ and photobio-electrochemical cells (PBEC).^40,41^ As outlined in Fig. 2, compared with the material-based biohybrids, these electrode-based systems have been more widely studied and understood in terms of their configuration and application.

Here, we will focus on the interactions between the biotic and abiotic elements in material-based cell hybrids, especially abiotic elements such as inorganic nanoparticles (NPs) through both chemical synthesis and biosynthesis, and conjugated polymers (nanoparticles) discussed in the following section. By reviewing the development of the materials as photocatalysts, we aim to not only provide the reason for choosing individual materials but also to suggest that conjugated polymers/NPs are promising candidates as light absorbers in semiartificial photosynthesis.

## 3. Chemical-synthetic inorganic nanoparticles: adsorption initiating the interactions

As an experimentally relatively simple approach presynthesised semiconductor particles have been combined with bacteria to give biohybrid systems. Several studies have reported hydrogen production for systems consisting of TiO_2_ and cells of a Gram-positive bacillus *Clostridium acetobutylicum*,^29^ cobalt phosphate, and genetically engineered *Saccharomyces cerevisiae*,^42^ or by using bismuth oxide or dye-sensitized TiO_2_ and the purple photosynthetic bacterium *Rhodopseudomonas capsulatus.*^43^ Upon irradiation, photoinduced electrons generated in the inorganic semiconductor NPs were transferred to microbes to produce hydrogen *via* redox couples. Recently, recombinant *Escherichia coli* cells expressing both hydrogenase and maturase genes have been reported to enable photocatalytic hydrogen production with TiO_2_ NPs acting as the semiconductors.^17^

Compared with purely artificial photocatalysts and enzyme-material-based systems, there has been a significant breakthrough in using whole-bacterial cells as biocatalysts. One of the greatest advantages is that the cells that serve as biocatalysts can be easily harvested from a liquid culture without the need for manipulations such as cell disruption and protein purification.^17^ Beyond this, the self-replicating nature of microorganisms grants cell hybrids potentially high scalability.^44^

**Fig. 3:**
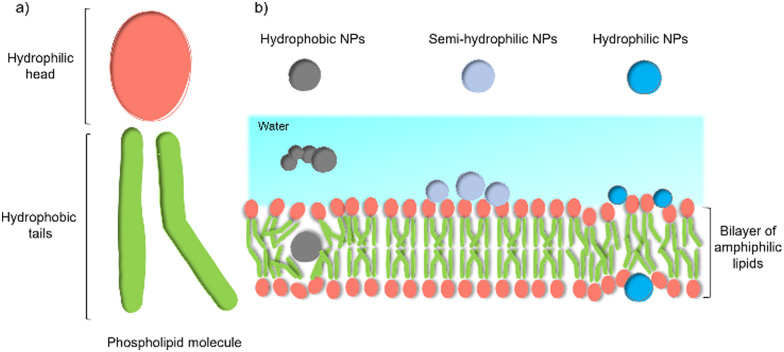
General structure of a membrane and the effect of NPs’ hydrophobicity/hydrophilicity on the interactions with the cell membrane.^47^ (a) An amphiphilic phospholipid bilayer consists of a hydrophilic head and hydrophobic tail. (b) Hydrophobic NPs prefer to embed themselves within the inner hydrophobic core of the bilayers. Semi-hydrophilic NPs tend to be adsorbed on the membrane surface.^48^ Large hydrophilic NPs (>20 Å) become wrapped and smaller ones (10 Å) embedded in the surface.^49^

Despite these benefits, a range of challenges must be overcome before the application in commercial solar energy conversion. Compared with MES or PBEC, cell-material-based hybrids are still in their infancy, and the nature of the interactions between the synthetic inorganic semiconductors and microbes in biohybrid photosynthesis systems is often not understood or even studied. Generally, the surface interactions of NPs with cells begin with adsorption.^45^ Three main factors affecting this process: (1) the NP's physio-chemical properties,^46^ such as size, shape, surface charge, and surface hydrophobicity/hydrophilicity; (2) cell type and physiological state;^47^ (3) experimental factors,^48^ such as adsorption temperature, ionic strength, pH, and osmolarity. This process is quite challenging because multiple factors can affect the interaction simultaneously. Among these factors, the hydrophobicity/hydrophilicity of the NPs play an important role.^49^ Several theoretical studies using a computational approach have demonstrated the effects of hydrophobicity/hydrophilicity on the interactions with cell membranes. Almost all biological membranes have a common structure: an amphiphilic phospholipid bilayer consisting of a hydrophilic head and hydrophobic tail (Fig. 3(a)).^50^ Molecular dynamic simulations revealed that the hydrophobic NPs are thermodynamically stable in the middle of the hydrophobic core of the membrane. In contrast, semi-hydrophilic NPs energetically prefer to be embedded in the bilayer surface (Fig. 3(b)).^51^ For the hydrophilic NPs with a diameter larger than 20 Å can be wrapped whereas those with a value of 10 Å will be embedded in the lipid bilayer.^52^ Therefore, for biohybrids of chemical synthetic NPs coupled with cells, it is difficult to determine the actual interactions between NPs and cells, and the absorbed NPs and suspended ones might co-exist in the colloidal biohybrid system. Another challenge is that many inorganic semiconductors studied so far contain heavy metals that can be cytotoxic to the organisms; it is important for this to be studied *in vivo* to ensure longer-term stability.^49^ Further modification of inorganic NPs, such as a multifunctional coordinating polymer coating,^53^ might provide one option that helps to alleviate the NPs’ toxicity to cells and to improve their cell-wall adsorption at the same time.

## 4. Biosynthetic inorganic NPs: bio-precipitation facilitating the connection

**Fig. 4:**
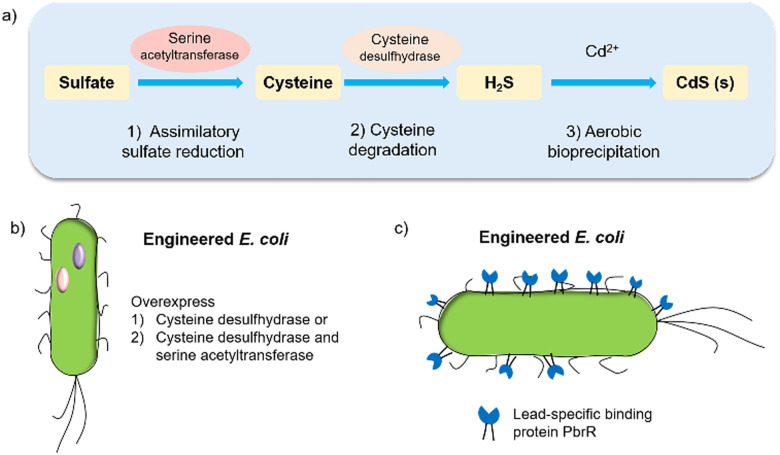
(a) CdS biosynthesis pathways in *E. coli*.^57^ (b) Representative genetic engineering method through overexpression of serine acetyltransferase and cysteine desulfhydrase. (c) Genetically engineered *E. coli* cell surfaces displaying the lead-specific binding protein PbrR.^15,59^

Biohybrid systems can also be obtained by using the biological organisms to facilitate the production of an artificial semiconductor within or on the organism. This approach has been used in particular for the synthesis of CdS nanoparticles.^54^ In particular, prokaryotic bacteria have been most extensively studied for the synthesis of inorganic materials. To produce nanocrystals, *E. coli* was incubated with CdCl_2_ and Na_2_S, to form CdS nanocrystals spontaneously.^55^ Furthermore, it is essential to prepare CdS directly in water for biological applications. Recently, the bio-precipitation of CdS NPs on the surface of the acetogenic, thermophilic bacterium *Moorella thermoacetica* (*M. thermoacetica*) successfully constructed an inorganic-cell hybrid system to produce acetic acid from carbon dioxide.^56^*E. coli* has also been used to drive the production of hydrogen with precipitated CdS on the cell surface.^19^*E. coli* produces cysteine from sulfides as part of its detoxification strategy through the expression of cysteine desulfhydrase, an aminotransferase that converts cysteine into pyruvate, ammonia, and hydrogen sulfide.^57^ This has been used by engineered *E. coli* to overexpress cysteine desulfhydrase to facilitate sulfide production and cadmium precipitation.^57^ Also, the gene encoding serine acetyltransferase was overexpressed for the production of cysteine (Fig. 4).^58^ In addition, *E. coli* mutants were constructed to express a lead-specific binding protein PbrR on the cell surface, permitting selective adsorption of both lead and cadmium ions and formation of PbS and CdS NPs on the cell membrane (Fig. 4(c)).^20,59^ These genetically engineered *E. coli* strains showed the impressive capability of facilitating the bio-precipitation of CdS and thus improving the hydrogen production of CdS-*E. coli* hybrids.

Other bacterial cells have also been used to synthesise gold nanoparticles. For example, intracellular synthesis of gold nanoparticles was achieved by using the mesophilic bacterium *Shewanella algae* with hydrogen as the electron donor.^60^ Recently, introducing gold nanoclusters into *M. thermoacetica* as light absorbers enabled the biosynthesis of acetic acid from CO_2_.^21^

The biosynthesis of metallic nanomaterials is another area of active research. There are several advantages to its application in semi-artificial photosynthesis. Potentially, the process provides a biocompatible light-harvesting inorganic semiconductor. Exogenous metal oxides, such as TiO_2_, are difficult to couple well with biocatalysts, which might reduce charge transfer efficiency and thus reduce solar-to-chemical conversion efficiencies. The close connection and structure of the biosynthetic nanoparticle–cell hybrid system enhance the electron transduction process.^19^ Several studies have shown that precipitation of CdS is typically associated with the bacterial cell wall, and occurs predominantly in the periplasmic space with the surface partially exposed to the extracellular environment.^19,55,61–63^ Thus, the extracellular photoelectron excitation and the cytoplasmic electron transduction process are improved.^19^ Combined with synthetic biology, cells can serve as a biofactory for various single- and multi-element nanomaterials,^64^ providing a platform for manufacturing inorganic semiconductors for semi-artificial photosynthesis. There have been numerous studies on the biosynthesis of nanomaterials, mostly including noble metals (Pd, Ag, Au, and Pt) and transition metals (Mn, Fe, Cu, Zn, Se, and Cd) in plant extracts, bacteria, fungi, and yeasts.^65–68^ The strategies developed will be useful for producing various inorganic semiconductors under mild conditions, serving as light absorbers.

## 5. Organic photocatalysts: moving from non-specific to specific interactions

The interface between the semiconductor and the cell appears to be crucial as it has a significant impact on the efficiencies of charge transfer events. As such, it is important to control physicochemical, molecular and cellular interactions of bacteria with the surface of the semiconductor. Important factors are hydrophobic interactions, surface charge, surface roughness, surface curvature, and the size and morphology of nanoparticles.^69^ Polymers are ideal for adsorption on bacteria due to their hydrophobic surface that reduces hydration and maximises adhesion.^70^ Adhesion can be further increased through the modification of the polymers with positively charged functional groups, such as quaternary ammonium groups. Increased electrostatic interactions are a result of the negatively charged outer membrane of bacteria. Surface roughness can be related to the increased surface area which in turn increases bacteria adhesion,^69^ but patterning of surfaces and submicrometric roughness has been shown to suppress the adhesion of bacteria.^71^ Following primary adhesion to the surface mediated by nonspecific interactions such as hydrophobic and electrostatic interaction^72^ irreversible adhesion takes place forming non-equilibrium hybrids.^73^

As discussed above, polymers have some benefits and can also be further tuned to bind to bacteria surfaces. This has been exploited to maximise selective and non-selective interactions. Specific biomolecular recognition units have also been utilized to obtain selective binding towards certain microbes, such as aptamers,^74^ anti-*E. coli* antibody,^75^ boronic acid derivatives,^76^ and mannose.^77^ The ability to copolymerize or functionalize polymers with the above recognition elements give rise to specificity. Other strategies include the grafting of polymers to surfaces,^78^ cell encapsulation,^79^ surface-initiated polymerisation,^80^ polymerisation inside cells, and templating of polymers on cell surfaces to maximise interaction.

**Fig. 5:**
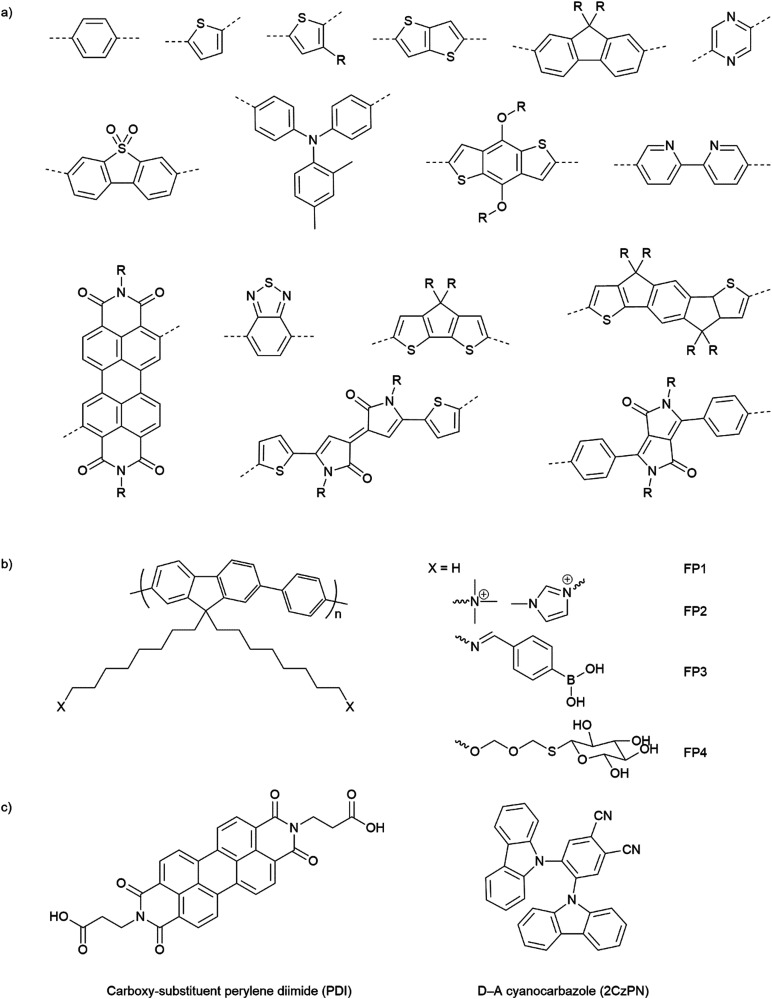
(a) Common building blocks in the synthesis of conjugated polymers. (b) Examples of side-chain modifications for conjugated polymers that could be used for assembly of biohybrids.^86,87^ (c) Reported molecule structures that were assembled into supramolecular nanostructures.^97^

Opto-electronic properties of organic molecules can also be tuned through synthetic methods. This can be used to obtain materials with strong and broad absorption in the visible region of the solar spectrum, also tuning of the band positions, charge separation, and charge migration efficiency.^12^ Furthermore, there are many widely available conjugated organic monomers, enabling synthesis of a broad range of conjugated polymers by well-established methods (Fig. 5(a)). As a result, organic photocatalysts, such as carbon nitrides (C_3_N_4_),^81^ carbon dots,^82^ conjugated microporous polymers (CMPs),^83^ covalent organic frameworks (COFs),^84^ and linear polymers,^85^ have recently been studied as photocatalysts.

Organic hybrid systems have been explored and recently, photosensitizers consisting of perylene diimide (PDI) derivative and poly(fluorene-*co*-phenylene) (PFP) were coated onto the surface of *M. thermoacetica* to create a biohybrid system for solar-to-chemical conversion.^22^ It was found that PDI/PFP interacted with the bacteria by electrostatic interactions and the cationic side chains of organic semiconductors intercalate into the cell membrane by hydrophobic interactions.^86^ Instead of coating organic materials onto bacteria cells, the microorganisms can also be immobilised onto the material surface.^26^ The study suggest that roughness appears to be the most crucial physicochemical property of the surface.^87^ Surface roughness increases the surface area available for bacterial attachment and provides a scaffold for adhesion.^88^ In addition, roughness can protect bacteria against shear forces resulting from flow in the cell culture during incubation or measurements.^89^ Compared with linear polymers, CMPs and COFs with porous structures and high surface areas show even greater potential in this respect. Microorganism immobilisation has yet to be demonstrated using CMPs and COF. However, enzyme immobilisation has been reported with COFs, which also raises another important property of the surfaces—the pore size. The reported chemically stable hollow spherical COF possesses mesoporous (2–50 nm) shells that provide adequate space for enzyme immobilisation.^90^ Apart from specific design and synthesis routes of CMPs and COFs, it was also shown that simple inorganic salts could alter the formation of micropores to macro–micro pores in CMPs,^91^ which might facilitate the microorganism immobilisation in biohybrid applications. As such, porous organic materials such as CMPs and COFs with high porosity and macropores/mesopores offer another opportunity for the assembly of biohybrids through the immobilisation of microorganisms.

The irreversible adhesion to surfaces by forming covalent bonds is another promising alternative. Metal–organic frameworks (MOFs) were reported to uniformly wrap *Morella thermoacetica* bacteria for cytoprotection in artificial photosynthesis. The MOF–bacteria interface involves direct bonding between phosphate units on the cell surface and zirconium clusters on the MOF monolayer.^92^ Recently, a “saccharide bridge” has been applied to promote the accumulation of conjugated polymer nanoparticles around *Pseudomonas aeruginosa*^93^ and *E. coli.*^94^ Adhesive bacteria such as *E. coli* can express type 1 pili where lectin domain FimH is the contributing adhesive site,^95^ and mannoside have been shown to bind adhesion FimH on *E. coli*.^96^ Phenylboronic acid (PBA) group in conjugated polymers can interact with the mannotriose or lactulose on the bacterial surface through the high affinity between diols and PBA,^97^ and mannotriose, as mannose derivative, can occupy adhesion sites of adhesive *E. coli*,^98^ both of which promote the specific attachment of conjugated polymers on the bacteria together. This strategy has been applied in the antiadhesive treatment of bacteria and sterilisation applications, and we believe that it could also provide insights into the assembly of material–bacteria hybrids in semi-artificial photosynthesis field. Taking fluorene-phenyl-based polymers as an example, compared with polymer FP1 and FP2 which mainly rely on nonspecific interactions, polymer FP3 with PBA groups (Fig. 5(b)) could improve the attachment to bacterial cells through both nonspecific and specific interactions; the latter one may also facilitate electron transfer between materials and microorganisms. Furthermore, instead of using PBA to interact with mannotriose that can occupy the adhesive sites on bacteria, mannose-substituted conjugated polymers,^99^ such as FP4, are also potential candidates in organic material-based biohybrids.

The conversion of bulk materials into nanosized structures may be necessary for the successful construction of organic material-based biohybrids because their much smaller size relative to bulk materials decreases the distance that has to be covered by photogenerated charges migrating to the surface of particles.^100,101^ The surface-to-volume ratio of nanoparticles is also greater than bulk materials, meaning that there is a larger interface available per unit mass. All other things being equal, both of these effects might be expected to increase photocatalytic efficiencies in nanohybrid materials *versus* those based on bulk particles. Compared with insoluble bulk polymers, soluble conjugated polymers open up opportunities for the processing of polymers into nanoparticles and films.^102^ Due to their inherent hydrophobicity, conjugated polymers can be self-assembled into polymer (nano)particles in water, mainly by mini-emulsion^103^ and antisolvent precipitation methods.^104^ The first reports on colloidal dispersions of nanoparticles of conjugated polymers appeared in the 1980s,^105^ and nanoparticles of conjugated polymers have gained attention only more recently in photovoltaics or biological imaging and cell labelling or photocatalytic hydrogen production.^106,107^ Recently, the conjugated polymer NPs were reported to bind to the outer membrane of *E. coli* cells through electrostatic attractions and hydrophobic interactions for cell imaging and barcoding.^108^ It was found that *E. coli* cells could be coated and tightened together with a continuous and coarse layer of conjugated polymer NPs.

Alongside the conjugated polymer NPs, synthetic supramolecular materials that have recently been applied for energy harvesting^109^ have provided another perspective into this field. Supramolecular materials comprise assemblies of molecular units that can form discrete entities or extended networks of both organic and hybrid nature.^110^ Supramolecular design and functions have been reviewed elsewhere^111^ and various molecules such as porphyrin,^112^ carboxy-substituent perylene diimide (PDI),^113^ and cyanocarbazole^114^ are referred to in Fig. 5(c). To our knowledge, synthetic supramolecular materials according to the definition above have yet been explored as light absorbers in semi-artificial photosynthesis. However, the ability to access various nanostructures (such as nanofibers,^113^ nanobelts,^115^ and nanoplates^116^) will enable coupling with microorganisms.

Compared to inorganic-based biohybrid systems, organic biohybrids have the advantage of leveraging assembly strategies that are not accessible with inorganic semiconductors. Organic semiconductors also have potential advantages due to their synthetic modularity across a wide range of commercial conjugated organic monomers, enabling the synthesis of a broad array of conjugated polymers through well-established methods (Fig. 5(a)). This allows the tuning of bandgaps and band positions, as well as strong and broad absorption in the visible region of the solar spectrum. It also offers a synthetic handle for important surface properties such as surface charge and wettability.^12^ In addition, organic semiconductors are carbon based which allows the materials to be designed to be closer in terms of physio-chemical properties compared to inorganic materials.^117^ This plays an important role in biohybrid systems as the surface of organic semiconductors is in the direct contact with biological systems, thus, it must be biocompatible with the surface.^118^ However, there are also drawbacks for organic materials due to intrinsic physical limitations. In particular, the high binding energies of excitons, which leads to low charge separation efficiencies at ambient temperature result in overall low apparent quantum efficiencies. Nevertheless, organic material biohybrid systems have not yet been studied extensively, and more fundamental studies are needed before conclusions on the long-term potential of organic biohybrids can be drawn.

## 6. Extracellular electron transfer mechanisms in hybrid photosynthesis

Following the construction of the hybrid system, the next step is to use and modulate the metabolic pathways of microorganisms to produce value-added chemicals: the presence of multiple metabolic pathways in biological organisms ensures the selective generation of metabolic molecules from water, CO_2_, and N_2_. An additional critical mechanistic requirement is the flow of electrons, to perform redox chemistry, and much research has gone into providing needed electron flow through direct and indirect electrical pathways. Initial research mainly focused on photocurrent production in microbial fuel cells (MFCs).^119^ In MFCs, a potential difference is created between the anode and cathode, and resistance is placed across an electrochemical cell to extract current from the microbial metabolism (Fig. 2(b)). Another application is bioremediation where metal-reducing microorganisms use metal as terminal electron acceptors such as Fe^3+^.^120^ Recently, microbial electrosynthesis (MES) reactors (Fig. 2(a)) have been studied as an alternative approach. They share similar characteristics with MFCs from the standpoint of circuitry, chamber layout, and basic electrochemical parameters,^39^ and apply current to the microorganisms to stimulate microbial metabolism for the reduction of CO_2_.

Extracellular electron transfer (EET) is a process in which microorganisms transport electrons into and out of the cell from or towards an insoluble electron donor or acceptor.^38,121^ The ability and efficiency of the organisms to exchange electrons with electrodes and more importantly to connect this EET to its original cellular metabolism is not only a crucial step in MFCs/MES but also a primary operational objective in the hybrid photosynthesis field. A more thorough understanding of possible EET pathways is needed to optimise and advance hybrid photosynthesis systems. Although there is abundant mechanistic information about microbially catalysed electron flow towards electrodes,^38^ not much is known about the biochemical mechanisms of EET from cathodes. This section discusses the possible strategies that organisms can employ to gain electrons from electrodes which might provide some consideration for material-based biohybrids in terms of EET.

### 6.1 Indirect extracellular electron transfer through hydrogen and electron shuttles

**Fig. 6:**
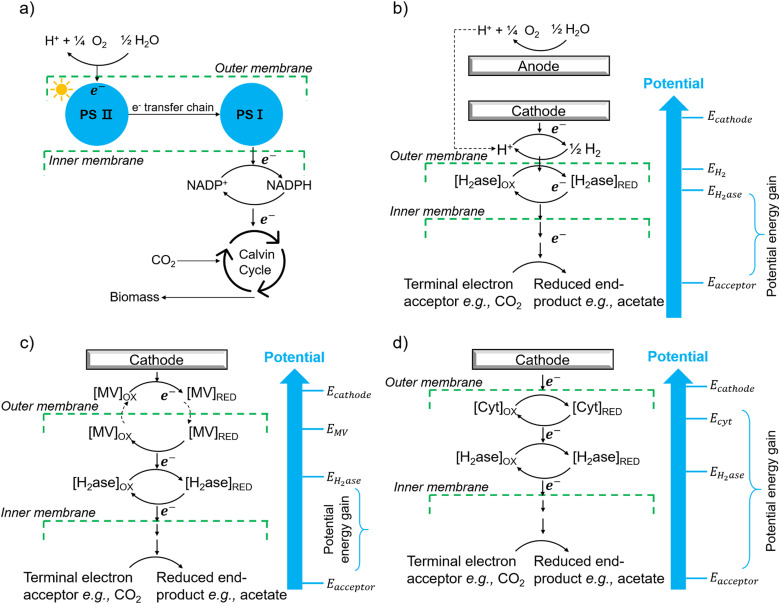
(a) The process of natural photosynthesis.^122^ (b) Proposed cathodic EET mechanism through hydrogen and associated microbial energy gains.^123^ (c) Mediated electron transfer through methyl viologen (MV) to a hydrogenase.^126^ (d) Direct electron transfer involving cytochrome-hydrogenase partnerships.^137^

The first potential extracellular electron transfer pathway is through hydrogen. The cathodic electrolysis of water to hydrogen can be exploited to deliver electrons to microorganisms indirectly. In a microbial electrosynthesis reactor, microorganisms accept reducing equivalents from an electrode in the form of H_2_ to reduce CO_2_ or other chemicals. In the hybrid photosynthesis field, the chemolithoautotrophic bacterium *Ralstonia eutropha* has been used for the aerobic production of bacterial biomass and liquid fuel alcohols.^122^ For instance, *Ralstonia eutropha* H16 oxidises hydrogen using hydrogenases to generate reduced cofactors (*e.g.* NADPH) and ATP, which are then used to reduce CO_2_ to 3-phosphoglycerate (3-PGA) *via* the Calvin–Benson–Bassham cycle. 3-PGA is then converted into biomass or may be diverted into isopropanol.^123^ This process mimics natural photosynthesis in which light harvesting, charge transfer, and catalytic functions are integrated to achieve solar-driven CO_2_ fixation (Fig. 6(a) and (b)).^124,125^ However, hydrogen-production approaches have several limitations: (i) the low solubility of hydrogen makes it difficult to achieve high local concentrations unless the microbial environment is pressurised; thus, microorganisms may have difficulties in efficiently consuming them;^126^ (ii) hydrogen production has increased energy costs due to the high overpotential required at non-catalyzed electrodes. Even when using a platinum-catalyzed cathode, the potential of the cathode will be lower than the theoretical standard electrode potential at pH 7, which is −0.410 V *versus* the standard hydrogen electrode (SHE).^127^*In situ* hydrogen generation by photocatalyst close to bacteria could be desirable to improve the efficiency and the energy cost of this route.

The second option of EET is through electron shuttles (Fig. 6(c)). Electron shuttles are redox-active molecules that can accept electrons from electrodes or other chemicals and delivery the electrons to microorganisms.^128^ Many stable redox shuttles have been used by bacteria to influence fermentation patterns or to promote the reduction of inorganic electron acceptors.^129,130^ Most commonly methyl viologen (MV^2+^), anthraquinone-2,6-disulfonate (AQDS), and neutral red (NR) have been used. For example, MV^2+^ has been used as the electron shuttle in the genetically engineered *E. coli* cells that produce the carboxysome shell-based nanoreactors to encapsulate oxygen-sensitive hydrogenases for hydrogen production;^130–132^ The advantages of electron shuttles involve that they can be dissolved at a higher concentration and typically be reduced at higher electrode potentials than hydrogen, thus saving energy.^129^ However, the current systems may have a high cost and need long-term stability,^133^ and the possible toxicity of many shuttles precludes their use in open environments and shuttles must be separated from products. It is also known that coloured redox shuttles can cause sacrificial loss of light that does not contribute to solar fuel production.

However, some microbially produced redox mediators provide another way to exchange electrons with electrodes, such as phenazines from *Pseudomonas* spp.^134^ and flavins from *S. oneidensis*,^135^ which represent important mediators for electron transfer between bacteria and from bacteria to anodes. It is likely that intrinsic redox mediators also play an important role in microbial biocathodes. Indeed, it was found that native pyrroloquinoline quinone^136^ could act as a reversible redox mediator for EET during biological oxygen reduction at a biocathode. Except for the self-excreted redox mediators, another possibility could be the secretion of whole enzymes that facilitate electron flow toward organisms. It has shown that cell-derived enzymes such as hydrogenases were released from *Methanococcus maripaludis* cells and accumulated at the cathode surface and the hydrogenase catalyzed the generation of hydrogen which in turn was immediately taken up by the cells.^137^ It has also been shown that native pyrroloquinoline quinone^136^ could act as a reversible redox mediator for EET during biological oxygen reduction at a biocathode. Except for the self-excreted redox mediators, another possibility could be the secretion of whole enzymes that facilitate electron flow toward organisms. It has shown that cell-derived enzymes such as hydrogenases were released from *Methanococcus maripaludis* cells and accumulated at the cathode surface and the hydrogenase catalyzed the generation of H_2_ which in turn was immediately taken up by the cells.^137^

### 6.2 Direct extracellular electron transfer involving c-type cytochromes and oxidoreductases

The third and, perhaps, most attractive method of achieving EET from electrodes is through direct electron transfer. Possible direct cathodic EET mechanisms have been studied, including the involvement of c-type cytochromes,^138^ the combination of c-type cytochromes and hydrogenases (Fig. 6(d)),^139^ the utilization of hydrogenase-containing microorganisms,^140^ or the employment of other oxidoreductases like copper-containing ones.^141^

*Geobacter* and *Anaeromyxobacter* species have been previously shown to accept electrons from graphite electrodes for the reduction of fumarate to succinate^142^ and nitrate to nitrite.^142^ Although *Geobacter* species have been shown to have many redox-active components on their outer membranes, including cytochromes and conductive pili,^143^ whether or not these could be used for accessing electrons in an oxidative systems remains to be explored.

Including earlier bioremediation of metal and organic contaminants, another exciting potential application of directly powering microbial activity is a microbial reduction of carbon dioxide with the release of extracellular, multi-carbon products. This form of microbial electrosynthesis is feasible with some acetogenic bacteria. For instance, the acetogen *Sporomusa ovata* formed biofilms on graphite electrodes and could accept electrons directly from the electrodes with the reduction of CO_2_ to acetate and small amounts of 2-oxobutyrate.^144^ Direct transfer was assumed to occur because the applied potential of the electrode was around −0.400 V *versus* SHE, which is higher than the previously described potential for H_2_ generation at graphite cathodes.^126^

**Fig. 7:**
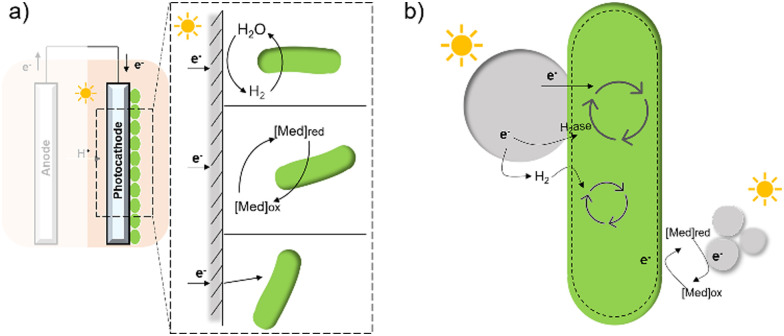
A summary of several hypothesised electron transfer pathways in electrode-based biohybrids^123,126,137^ (a) and photocatalyst-based biohybrids (b) including methods through hydrogen, redox mediator, and direct transfer.^12,14,127^

In contrast to electron transfer between electrodes and microbes (Fig. 7(a)), much less is known about the mechanism by which the cell takes up electrons from the photocatalysts in the field of hybrid photosynthesis. Several potential electron transfer pathways of photocatalysts-based hybrids have been proposed, and some similarities in the EET could be found in electrode hybrids (Fig. 7(b)). In TiO_2_/*E. coli* hybrids that were developed for photocatalytic H_2_ production, MV^2+^ was used as a redox mediator to shuttle electrons between microbes and inorganic photocatalysts.^17^ Notably, H_2_ formation was detected even in the absence of MV^2+^, indicating that electrons may also be transferred directly from the conduction band of TiO_2_ to the microorganism.^17^ Additionally, biosynthetic materials, like CdS,^19,56^ were reported to be constructed cell–material hybrid systems without the addition of electron transfer agents. Furthermore, a dual pathway was proposed through transient absorption spectroscopy studies: a non-hydrogenase-mediated pathway and a membrane-bound hydrogenase-mediated pathway.^145^

### 6.3 Factors involved in extracellular electron transfer

**Fig. 8:**
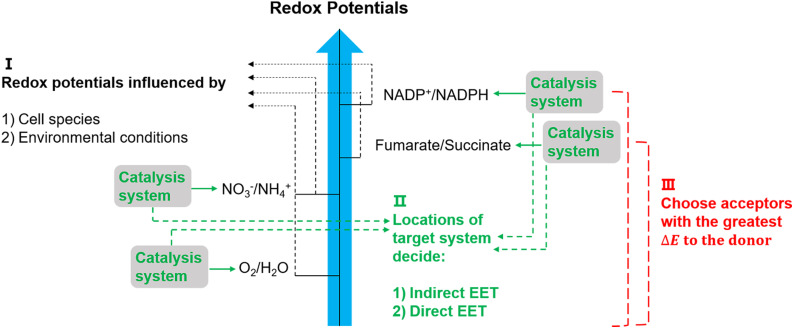
Potentially relevant factors for EET pathways. Cell species and environmental conditions, *e.g.* pH, influence the potentials of redox reactions, and the locations of the systems that used to catalyse these reactions might result in an indirect EET or direct EET.^144,145^ Lastly, microorganisms have evolved to have as large as possible redox potential differences to maximise energy gains.^144^

The suggested electron transfer pathways discussed above could be considered as electric interactions between electrode/material and microbes, and several factors such as the target site of EET, bacterial species, and environmental conditions are involved in this process (Fig. 8).

The location of the target site decides if an organism will perform direct or indirect EET. It was proposed that microbes with outer membrane redox-components could perform direct EET while organisms feature soluble intracellular complexes such as hydrogenases of acetogens are more likely to conduct indirect EET *via* mediators.^146^ Additionally, the bacterial species and environmental conditions such as pH, redox potential of the solution, and specific concentrations of redox couples could affect the required redox potentials in EET,^147^ and thus could influence the available energy gained through EET. Since the achievable energy yield of each electron transfer pathway relies on the difference in redox potential (Δ*E*) of all redox reactions between electron donor and acceptor, microorganisms will always choose available acceptors with the greatest potential difference to the donor.^146^

In the electron transfer pathways of microbial cells, electrons are transferred from a low potential electron donor to a higher redox potential electron acceptor through redox reactions. These reactions are usually catalyzed by important systems that use the energy difference between the donor and acceptor to establish an ion gradient across the membrane, such as Na^+^ and H^+^.^148^ These systems include dehydrogenases and transmembrane electron transporters including cytochromes and terminal oxidoreductases.^148^ Also involved are electron-carrying cofactors such as quinones, flavins, heme, iron–sulfur clusters, or copper ions to shuttle electrons between large enzymatic complexes inside the membrane.^146^ The ion gradient established by those systems is a motive force across the membrane that can drive ATP synthesis.^149^ This is not only a significant factor for bacterial growth but also crucial for metabolic pathways that require energy.^150^

**Fig. 9:**
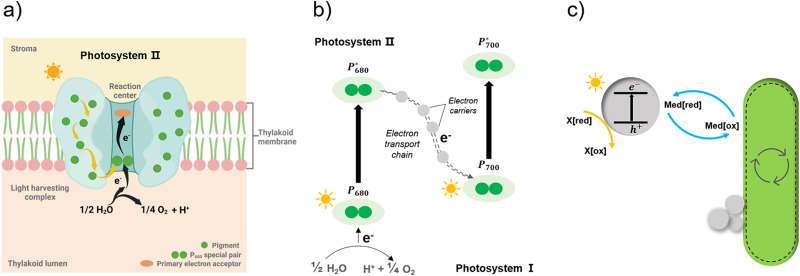
Simplified illustration of electron generation and transport in natural photosynthesis and biohybrid system through redox mediators.^149^ (a) and (b) Light absorption and electron transfer in photosystem II. When one of the pigments in light harvesting complex is excited by light, energy is transferred from pigment to pigment through resonance energy transfer until it reaches the reaction centre. There, P680 special pair is excited and loses an electron, passing it to primary electron acceptor in the complex and replaced with an electron from water. With this transfer, the electron begins its journey through an electron transfer chain *via* electron carriers to photosystem I. Created with BioRender.com. (c) In biohybrid system, when semiconductors are excited by light electrons are transferred *via* redox mediators to bacteria cells and replaced with electrons from sacrificial electron donors.

Apart from the factors discussed in Fig. 8, the role of redox mediators and sacrificial electron donors (SED) applied in the biohybrid systems must be studied as well. As shown in Fig. 9(a) and (b), electrons are transferred from PS II to PS I *via* electron carriers in the electron transport chain and replaced with electrons from water.^151^ In biohybrid systems, redox mediators or electron shuttles such as MV^2+^ are normally used. Involving a redox mediator in the biohybrid systems is useful to suppress the charge recombination between photosensitizer and bio-catalytic sites. However, the diffusion of the redox couple has a significant influence on the electron transfer rates in biohybrid systems. When polymer materials are used as photosensitizers, the interaction and thus charge-transfer event between the redox mediator and the polymer matrix may be the rate-determining step. Using a high concentration of redox mediators is an effective solution to overcome this. However, the redox mediator such as natural red and reduced MV^2+^ can also result in competitive light absorption with the photosensitizer. The role of the SED such as cysteine or ascorbic acid in the biohybrid systems must also be considered (Fig. 9(c)), as it might also determine the charge transfer pathway, thus influencing the performance and stability of biohybrid systems. For example, if the excited photosensitizer undergoes oxidative quenching by the redox mediator, then the SED will determine the regeneration rate of the photosensitizers, thus having a significant effect on the stability of photosensitizer; however, if the excited photosensitizer needs reductive quenching from the SED first, then the electron transfer from the SED to the photosensitizer could become the rate-determining step in the entire photocatalytic processes.

Without a deeper understanding of the underlying electron transport mechanism, the development of hybrid photosynthesis will remain a trial-and-error exercise. Therefore, it is of great importance to reveal the fundamental EET process to optimise and advance different envisioned applications in hybrid photosynthesis. Spectroscopic techniques,^145^ such as time-resolved single-photon counting, transient absorption spectroscopy, and time-resolved microwave conductivity should be studied more in the context of these systems to give insight into the nature of charge carriers and their kinetics/transfer rates.

## 7. Using organic polymers as energy transfer antennas between photosensitizer and bio-catalyst

Besides electron transfer from the photosensitizer to the bio-system to perform photocatalysis, energy transfer could also be an important route to enhance photocatalytic reaction, particularly for those photosynthetic bacteria. Wang, Liu, and co-workers^152^ proved that coating chloroplasts with conjugated polymer NPs consisting of poly[2,7-(9,9-di-*n*-hexylfluorene)-*co*- (*para*-phenylene)] (PFP) and poly[(9,9-di-*n*-octylfluorenyl-2,7-diyl)-*co*-(1,4-benzo-{2,1′,3}-thiadiazole)] (PFBT) can enhance the light-harvesting by chloroplasts due to efficient energy transfer from polymer NPs to chloroplasts. This strategy provides the potential use of polymer materials as energy antennas for photosynthetic bacteria. As the energy transfer is highly dependent on distance, realising Förster energy transfer or Dexter energy transfer requires proximity between the photosensitizer and the biological light harvester, *e.g.*, chloroplasts. Whatever process is used for the energy transfer, polymers or polymer NPs require high emission quantum yields and must be matched in terms of their emission profiles to the absorption profile of the biological light harvester system.

## 8. Summary and outlook

A range of different methods has been used to assemble semi-artificial systems. We believe that organic photosensitizers, especially conjugated polymers, are promising because they can provide specific interactions with the cell surfaces, which could yield important breakthroughs in terms of performance. To achieve this, it is important to study a wider range of organic materials classes and their different forms (*e.g.*, soluble polymers, insoluble CMPs and COFs, nanoparticles, crystalline and amorphous analogues). It is still unclear what the optimal materials are in terms of polymer backbone structure. It is also unclear which surface interactions should be prioritized to obtain high-performing hybrid systems, although there is a growing body of literature to support this choice. The use of specific binding units, such as aptamers, antibodies, boronic acid derivatives, and sugars remains unexplored so far in this field. The use of automation and robotics might greatly accelerate the testing of large numbers of semiconductors. The potential of such approaches was shown for abiotic photocatalyst discovery^153^ and optimization,^154^ and for biohybrid systems, the potential combinatorial space is even larger. These strategies could be expanded to include, for example, dye-sensitization strategies and the addition of mediator components that might enhance charge transfer processes.

There is also an opportunity to use new, unexplored methods: strategies such as the grafting of polymer sensitisers to surfaces, cell encapsulation with polymers, surface-initiated polymerization, polymerization inside cells, and templating of polymers on cell surfaces all remain unexplored in the context of photocatalytically active hybrid systems. Porous organic materials offer also the potential to increase the surface area and thus to interact more effectively with cells, and this is an area that has not been studied so far. Solution processible materials can be cast into films that then can be used in hybrid systems. This might have advantages for scale up and here cell colonies and biofilms might also be studied, which could possess greater stability and even the ability to regenerate. As in abiotic photocatalysis, there is little standardization between laboratories, with different equipment being used in each study, making it hard to compare performance of different systems directly. It would therefore also be highly desirable to standardize performance metrics, for example by reporting quantum efficiencies and solar-to-fuel efficiencies using reproducible (and ideally, standardized) protocols.

This area will not advance using methods such as automation and robotics alone. More effort should be made to gain an understanding of the underlying fundamental processes in these biohybrid systems. In particular, electron transport chains and intracellular electron transport should be studied in more detail, and spectroscopic techniques, such as transient absorption spectroscopy coupled with metabolic analysis, should allow further insight. Spectroscopic techniques will not only assist in studying potential charge transfer mechanisms but also provide the opportunity to distinguish various dominant electron/energy transfer mechanisms at different timescales.^145^ This will also be beneficial when it comes to the fabrication of devices that rely on contact with external circuits, such as electrode-based biohybrid photocatalytic systems, which currently suffer from poor charge collection efficiency from the counter photoelectrode.

Finally, other more challenging reactions, such as CO_2_ reduction and N_2_ fixation,^155,156^ offer opportunities for biohybrid systems. Given the evolved ability of natural systems to activate carbon dioxide and nitrogen products, it might be possible to obtain products that are currently not accessible using artificial photocatalysts.

## Conflicts of interest

There are no conflicts to declare.

## Supplementary Material
